# A Machine Learning Approach to Predict *Listeria monocytogenes* Growth by Integrating FSSP and PMP Simulations: A Focus on *t*_100_ as a Possible Endpoint Parameters for Predictive Models

**DOI:** 10.3390/foods15203640

**Published:** 2026-10-14

**Authors:** Mario Terlizzi, Barbara Speranza, Angela Racioppo, Milena Sinigaglia, Maria Rosaria Corbo, Clelia Altieri, Antonio Bevilacqua

**Affiliations:** Department of the Agriculture, Food, Natural Resources and Engineering, University of Foggia, Via Napoli 25, 71122 Foggia, Italy; mario.terlizzi@unifg.it (M.T.); barbara.speranza@unifg.it (B.S.); angela.racioppo@unifg.it (A.R.); milena.sinigaglia@unifg.it (M.S.); mariarosaria.corbo@unifg.it (M.R.C.); clelia.altieri@unifg.it (C.A.)

**Keywords:** food safety, random forest, machine learning, *t*_100_, predictive microbiology

## Abstract

*Listeria monocytogenes* is one of the most challenging foodborne pathogens for its psychrotrophic nature as well as the ability to grow under a wide range of environmental conditions; thus, food producers and regulatory agencies always search for new strategies to reduce the risk. In this context, predictive microbiology represents a valuable tool for supporting risk assessment and food safety management. This study investigated the time required for a 2-log increase in *L. monocytogenes* population (*t*_100_) as an integrated time-to-threshold endpoint and developed a simulation-derived modeling framework combining outputs from the Food Spoilage and Safety Predictor (FSSP) and Pathogen Modeling Program (PMP). After a preliminary analysis based on DoE (Design of Experiments) theory, the dataset was expanded with 300 additional simulations and used to compare different modeling approaches (e.g., nonlinear models and Random Forest regression). The optimized Random Forest showed the best internal predictive performance; temperature and NaCl were the dominant predictors, followed by nitrite and pH, whereas simulation source and initial inoculum showed lower additional contributions. A separate FSSP-specific analysis showed that the inclusion of lactic acid, an input variable of this tool, did not improve the predictive performance of the model. Concerning the accuracy of the model, it is worth mentioning that error increases for high *t*_100_ values and near the boundaries of the represented environmental space. Nevertheless, *t*_100_ provides a useful integrated endpoint for combining heterogeneous simulated growth responses. However, the proposed model should be regarded as a simulation-derived metamodel whose performance has been assessed only by an internal cross-validation. Independent validation through challenge tests in representative food matrices is required before its applicability to practical food-safety decision making.

## 1. Introduction

Predictive microbiology represents a major shift in the food sector, moving the approach from traditional laboratory testing to a proactive, quantitative approach using mathematical models to predict how microorganisms behave based on environmental conditions. The modern discipline grew between the late 1980s and early 1990s. Ratkowsky et al. [1] established the first links between temperature and bacterial growth rates; then, Zwietering et al. [2] standardized the description of microbial growth using the modified Gompertz model.

Predictive microbiology helps regulators and industries to perform quantitative risk assessments [3], allowing users to simulate critical scenarios, such as power failures during transport, thus reducing the need for expensive and slow “challenge tests” [4]. Today, the field is moving toward “Microbiology 4.0”, as noted by Taiwo et al. [5], as researchers are now integrating AI and genomic data. This leads to more precise models that can predict the behavior of specific microbial strains in complex food products.

In this regard, the use of predictive microbiology is essential for studying the behavior of *Listeria monocytogenes*, one of the most significant pathogens in food safety. Its importance stems from the severity of listeriosis and the organism’s high environmental resistance [6]. Unlike many other bacteria, *L. monocytogenes* is a ubiquitous and psychrotrophic pathogen. This means it can survive and grow at refrigeration temperatures, making the cold chain an insufficient safeguard. Furthermore, the pathogen demonstrates an extraordinary ability to develop under adverse conditions, tolerating high salt concentrations and wide pH ranges [7]. These traits pose a constant risk to Ready-to-Eat (RTE) products, which are consumed without prior heat treatment.

In the European Union (EU), the institutional management of this hazard is based on Regulation (EC) No 178/2002 [8], which established risk analysis as a fundamental regulatory pillar. This legislation marked a shift from inspecting finished products to the proactive responsibility of Food Business Operators (FBO), as they must guarantee safety throughout the entire production chain. Subsequent regulatory updates introduced stricter microbiological criteria through Regulation (EC) No 2073/2005 [9]. Finally, Regulation (EU) 2024/2895 [10] introduced significant innovations that are fully mandatory starting July 1, 2026. In this regulatory context, predictive microbiology has become an essential decision-making tool for risk assessment and process validation [11]. Mathematical models allow for the simulation of *Listeria*’s kinetic behavior in response to variables such as temperature, pH, and water activity. This “in silico” approach enables FBOs to reduce analytical costs and manage risks dynamically [12], provided it is supported by adequate training and intuitive software interfaces. The true value of these systems lies not in their mathematical complexity, but in their ability to translate that complexity into practical outputs, such as residual shelf-life estimates [13]. Making these tools more accessible is precisely where recent advances are heading, as new modeling methodologies in food safety are increasingly influenced by integration with AI [14].

Machine learning algorithms are becoming essential components of these evolving models [15]. By leveraging these technologies, researchers can quickly and effectively analyze and interpret complex datasets to derive meaningful insights. These modeling tools find application across various areas of food safety, particularly in risk assessment and proactive management [5]. The integration of AI and machine learning is driving the transition toward “Predictive Microbiology 4.0”. Unlike classic models, AI algorithms like Neural Networks and Random Forests can identify complex, and nonlinear relationships between environmental variables [16]. Furthermore, combining AI with Whole Genome Sequencing (WGS) enables the development of strain-specific models, significantly improving prediction accuracy for pathogens like *Listeria monocytogenes* [17]. This digital transition, supported by loT and cloud computing, allows for real-time monitoring and proactive decision-making across the food supply chain [5].

Therefore, the aim of this study was to propose a first approach in developing an original third-level predictive model to estimate *t*_100_ of *L. monocytogenes*, that is, the time to gain a 2-log increase, by integrating the traditional predictive microbiology with the machine learning approach. Using an integrated approach of primary, secondary and tertiary models, simulated data were analyzed to identify statistically significant relationships between environmental variables and microbiological outputs. As a secondary goal, different modeling approaches, specifically nonlinear logistic models and Random Forest algorithms, were compared to test their suitability in the context of the Microbiology 4.0.

## 2. Materials and Methods

### 2.1. Methodological Approach

The starting point of this research was the development of a multivariate experimental design based on the Design of Experiment (DoE) theory. The objective was to analyze the simultaneous influence of various environmental factors (temperature, pH, NaCl, nitrites, and lactic acid) and initial microbial concentration on the growth of *L. monocytogenes*. These combinations were simulated using FSSP version 4.0 and PMP online tool. Finally, the simulation outputs were processed via dynamic modeling using the Gompertz equation, as modified by Zwietering et al. [2] and by Corbo et al. [18], to estimate the *t*_100_ value and the other fitting parameters (growth rate and lag phase), which were then used as the main input for a secondary modeling approach. After the choice of the kinetic parameter for the development of the regression/Random Forest model other 300 additional combinations from FSSP and PMP were employed and the relative fitting parameters estimated. The resulting dataset (data from the first and the second step) was used for the modeling approach (nonlinear models and Random Forest).

### 2.2. Simulation of Listeria monocytogenes Growth Using FSSP Software: First Phase

The simulation by Food Spoilage and Safety Predictor (FSSP) focused on *L. monocytogenes* growth in chilled foods, with a maximum storage period of 90 days. The study was based on an experimental design featuring six independent variables, each set at three levels (−1, 0, +1), as detailed in Table S1.

For each combination, the software provided the following output parameters: specific growth rate (µ, h^−1^); lag phase duration (days); time to reach the critical threshold (*t*_100_), defined as the time required for a 2-log CFU/g increase.

The full set of experimental combinations and simulation results is provided in Table S1.

### 2.3. Experimental Design and Simulation Using Pathogen Modeling Program (PMP)

Then, Pathogen Modeling Program software developed by the United States Department of Agriculture (USDA) was used. To systematically investigate factor interactions, a Central Composite Design with five variables was implemented.

The “Growth anaerobic (NaCl)” model was selected for simulations to represent the behavior of *L. monocytogenes*. The simulation duration was set to 200 h to capture the full microbial growth cycle; for each combination, the software provided the following output parameters: growth rate; lag time; maximum population density.

The final dataset, integrating experimental inputs and predictive outputs, was organized in Table S2 of the Supplementary Materials.

### 2.4. Microbial Growth Modeling Using the Gompertz Equation

To describe the growth kinetics of the 47 CCD-generated combinations, data obtained from PMP (mean, LCL, and UCL values, that is lower and upper limits of confidence interval) were modeled using Gompertz equation in both its standard and reparametrized forms. This approach allowed for both the precise estimation of the sigmoidal curve parameters and the prediction of the *t*_100_ value, which is not directly provided as an output by PMP.

Modeling was performed using Statistica for Windows, ver. 14.0 (Nonlinear Estimation module) (TIBCO Software Inc., Palo Alto, CA, USA) via User Specified Regression. The parameters were defined as follows:

- *b*_0_: initial microbial concentration;
- *b*_1_: microbial concentration increases at the stationary phase;
- *b*_2_: maximum growth rate;
- *b*_3_: lag phase duration;
- *b*_4_: time to reach the critical threshold (Lc, set as a 2-log CFU/g increase).
  The equations implemented in the software were:
- Classic Gompertz [2]:
  $$y=b_{0}+b_{1}\cdot exp(-exp(((b_{2}\cdot e)\cdot (b_{3}-time)/b_{1})+1))$$
- Reparametrized Gompertz [18]:
  $$y=Lc-b_{1}\cdot exp(-exp(((b_{2}\cdot e)\cdot (b_{3}-b_{4})/b_{1})+1))+b_{1}\;\cdot exp(-exp(((b_{2}\cdot e)\cdot (b_{3}-time)/b_{1})+1))$$

### 2.5. DoE Statistics

The kinetic parameters from FSSP and PMP were modeled through the option DoE of the software Statistica for Windows; the statistical weights of the variables (temperature, NaCl, initial concentration, etc.) were assessed through the Pareto chart, while contour plot and desirability profiles were used to describe the quantitative effects of the factors on the kinetic parameters [19]. Desirability profiles were generated separately for each kinetic response; the function was scaled between 0 (least desirable response) and 1 (most desirable response).

The desirability profile was evaluated as follows, with *y_min_* and *y_max_* being the minimum and the maximum values of the dependent variables:

$$d=\left\{\begin{array}{ll} 0, & {y\leq y}_{min}\\ {(y-y}_{min})/(y_{max}-y_{min}) & y_{min}\leq {y\leq y}_{max}\\ 1, & {y\geq y}_{max} \end{array}\right.$$

### 2.6. Second Simulation Procedure. Second Phase

A second simulation process on FSSP and PMP was performed to expand the dataset and provide a broader and more heterogeneous set of environmental conditions for model development. A total of 300 additional combinations were generated, including 150 simulations with FSSP and 150 with PMP (Supplementary Tables S3 and S4).

For each additional combination, simulations were performed according to the procedures described in Section 2.2, Section 2.3 and Section 2.4. The input variables included temperature, pH, NaCl concentration, nitrite concentration, initial *L. monocytogenes* concentration, and lactic acid only for FSSP simulations. The newly generated simulations were then combined with those obtained during the first simulation process to construct the expanded dataset used for predictive modeling (Section 2.7).

### 2.7. Development of a Unified Model

The expanded dataset was used to develop predictive models using *t*_100_ as the response variable. Before modeling, initial cell concentration was reported for all the combinations as log CFU/g, while time units were all reported in days. First, a nonlinear logistic model based exclusively on the common environmental predictors was fitted as a reference model. The general formulation was:

$$t_{100}=\frac{a}{1+exp\left(\eta \right)}$$

where *a* is the upper asymptote and *η* a liner predictor, calculated through the following formula:

$$\eta =\beta_{0}+\beta_{1}T+\beta_{2}pH+\beta_{3}NaCl+\beta_{4}nitrite+\beta_{5}concentration$$

For a second model, simulation source was introduced as an additional predictor to account for possible systematic differences between FSSP and PMP. The linear predictor was as follows:

$$\eta =\beta_{0}+\beta_{1}T+\beta_{2}pH+\beta_{3}NaCl+\beta_{4}nitrite+\beta_{5}concentration+\beta_{6}S$$

where *S* is binary indicator of simulation source, set as follows: FSSP = 0; PMP = 1.

In the third model, interactions between simulation source and environmental predictors were considered. The equation of linear predictor was:

$$\eta =\beta_{0}+\sum_{j=1}^{5}\beta_{j}X_{j}+\beta_{6}S+\sum_{j=1}^{5}\gamma_{j}\left(S*X_{j}\right)$$

where *X_j_* represents the five common quantitative predictors (T, pH, NaCl, nitrite, concentration), *β* their main effects, and *γ* the source-dependent interaction terms.

Finally, Random Forest regression was evaluated. For an input vector, defined as *X* = [T, pH, NaCl, nitrite, concentration, S], the Random Forest prediction was defined as the average prediction generated by the individual regression trees:

$$t_{100}\left(x\right)=\frac{1}{B}\sum_{b=1}^{B}T_{b}\left(x\right)$$

where *T_b_*(*x*)_is the prediction of the *b*-th regression tree and *B* is the total number of trees in the ensemble.

For each modeling approach, performance was evaluated using the coefficient of determination (R^2^) and root mean squared error (RMSE).

Model performance was internally assessed using 5-fold cross-validation. The dataset was randomly divided into five approximately equal subsets. At each iteration, four folds (approximately 80% of the observations) were used for model fitting and the remaining fold (approximately 20%) was used for validation. This procedure was repeated five times so that each observation was used once for validation.

### 2.8. Assessment of the Effect of Lactic Acid

Because lactic acid concentration was available only for FSSP simulations, this variable could not be included directly in the integrated FSSP–PMP. Its potential contribution was therefore evaluated separately using the FSSP subset. Two Random Forest models, respectively excluding and including lactic acid concentration, were compared using repeated 5-fold cross-validation. The approach was that reported in the Section 2.7.

### 2.9. Model Optimization

The structural hyperparameters of the Random Forest were optimized using a randomized search procedure. The search evaluated maximum tree depths of 8, 10, 12, 16, 20, or unrestricted depth; the proportion of predictors considered at each split (max_features) was tested at 0.50, 0.70, 0.85, 1.00, or the square root of the total number of predictors. Values from 1 to 4 were explored for min_samples_leaf and from 2 to 8 for min_samples_split. Sixteen randomly selected hyperparameter combinations were evaluated using shuffled 5-fold cross-validation, with RMSE as the optimization criterion. A fixed random seed (20260919) was used to ensure reproducibility.

After selection of the structural hyperparameters, the number of trees was set to 1000 for final model fitting to stabilize ensemble predictions. R^2^ and RMSE were calculated for each validation fold and summarized across repetitions. In addition, out-of-bag (OOB) performance was calculated for the Random Forest fitted to the complete modeling dataset as an additional internal estimate of predictive performance. Predictor contribution was quantified by permutation importance, i.e., by measuring the decrease in predictive performance following random permutation of the values of each predictor while leaving the remaining variables unchanged.

To determine whether predictive error was uniformly distributed across the response domain, cross-validated predictions from the optimized Random Forest were additionally analyzed. Observations were divided into quartiles of the *t*_100_ distribution, evaluating for each quartile RMSE, mean absolute error (MAE), and mean residual

### 2.10. Domain Analysis of Random Forest Model

The 10th and 90th percentiles (P10 and P90) of temperature, pH, NaCl concentration, nitrite concentration, and initial cell concentration were calculated separately for each simulation source (FSSP and PMP), and the combinations were thus divided into interior (if all the predictors were in the range P10–P90) and boundary if at least one predictor was outside the range. For these two new sets of combinations, Random Forest was run again and RMSE, MAE, and mean residual calculated.

### 2.11. Statistics

DoE analyses (Pareto charts, desirability profiles, and response-surface analyses) were performed using Statistica for Windows. Predictive modeling and machine learning analyses were performed using Python 3.13.5 (Python Software Foundation, Wilmington, DE, USA) and the scikit-learn 1.8.0 library (scikit-learn developers). Data preprocessing and dataset management were performed using pandas 2.2.3 and NumPy 2.3.5, while graphical representations were generated using Matplotlib 3.10.8. Random Forest regression, cross-validation, randomized hyperparameter optimization, permutation importance, and calculation of model-performance metrics were implemented using scikit-learn. Fixed random seeds were used where applicable to ensure reproducibility of the analyses.

## 3. Results

### 3.1. Desirability Profiles from FSSP Simulations

**Figure 1:**
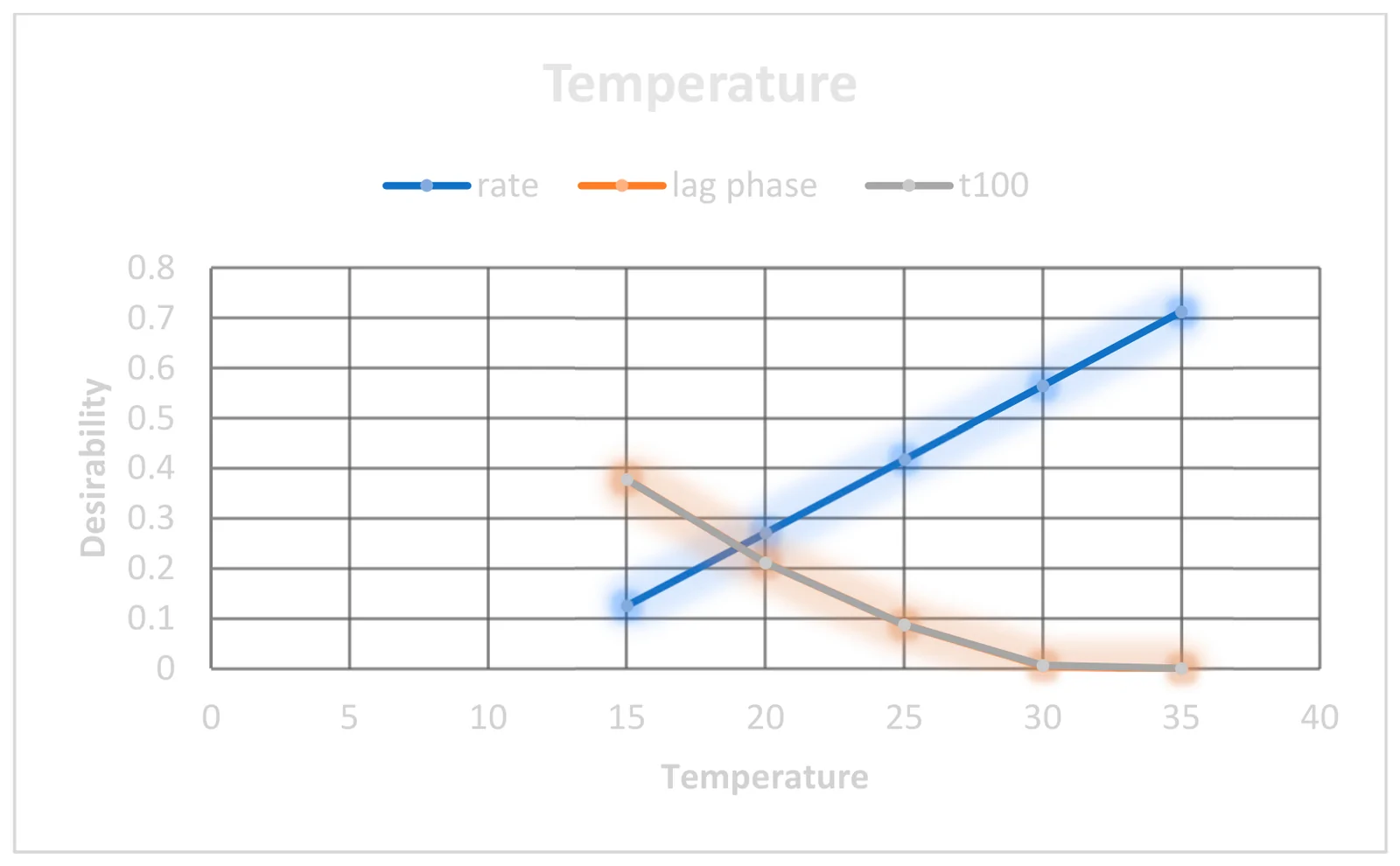
Temperature desirability profiles for kinetic parameters estimated with FSSP.

Desirability function was used to compare the statistical effect and the weight of each input factors on the fitting parameter of *L. monocytogenes*. To improve the readability and also for comparative purposes, for each factor (temperature, pH, NaCl, etc.) the desirability was extracted to build a comprehensive profile combining all kinetic parameters. Temperature showed the most pronounced influence; as temperature increased, a clear increase in the specific growth rate and a progressive decrease in both the lag phase and the *t*_100_ parameter were observed. In the corresponding desirability plots in Figure 1, the curves for the lag phase and *t*_100_ are almost perfectly overlapped. This behavior suggests that, within the experimental domain, variation in *t*_100_ is largely driven by variation in the lag phase.

**Figure 2:**
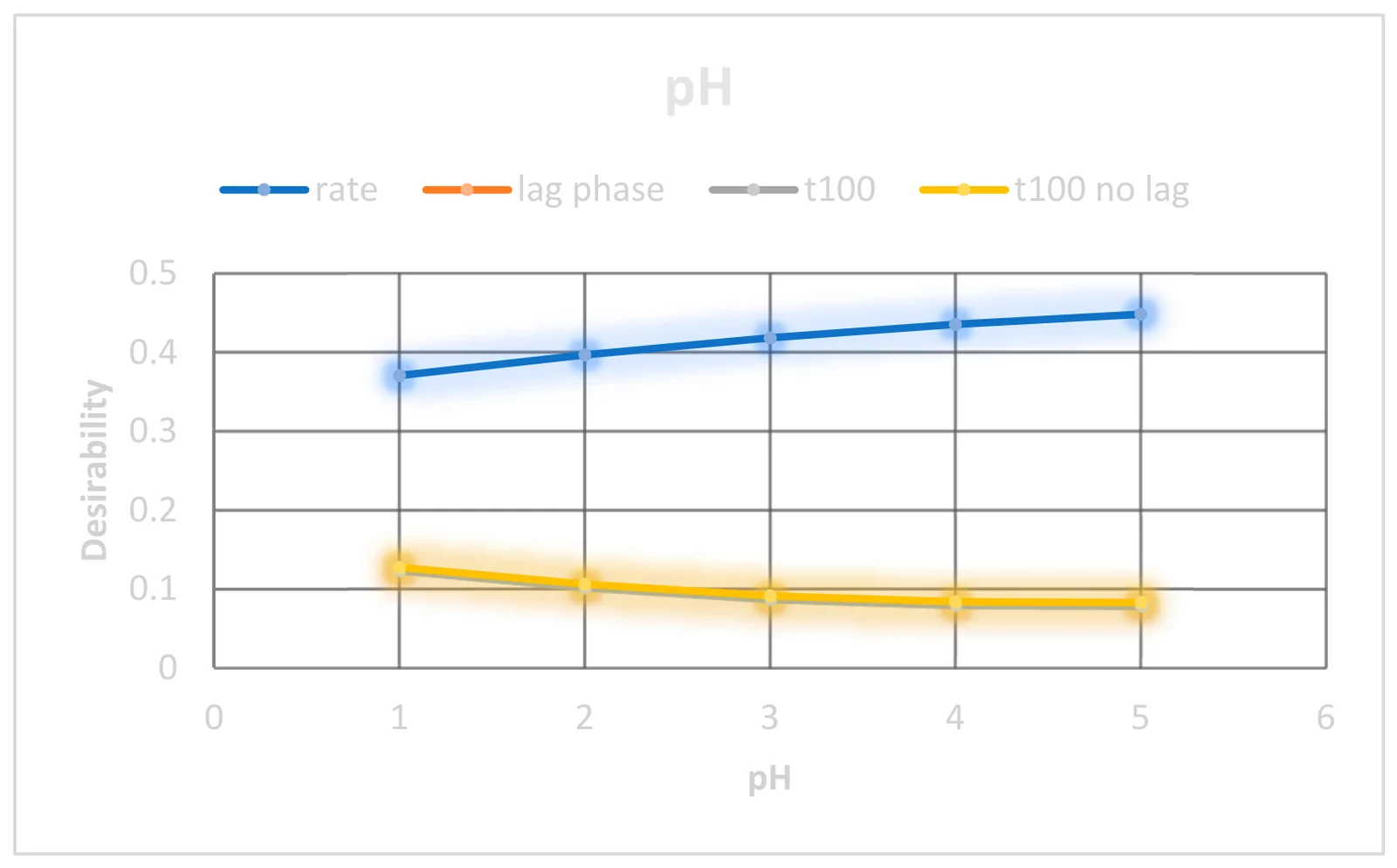
pH desirability profiles for kinetic parameters estimated with FSSP.

On the other hand, pH shows a strong optimizing behavior as values increase, with a nearly linear correlation (Figure 2). Moving from acidic conditions to sub-alkaline values, the desirability of the growth rate increases, which simultaneously causes a clear reduction in lag times and *t*_100_.

**Figure 3:**
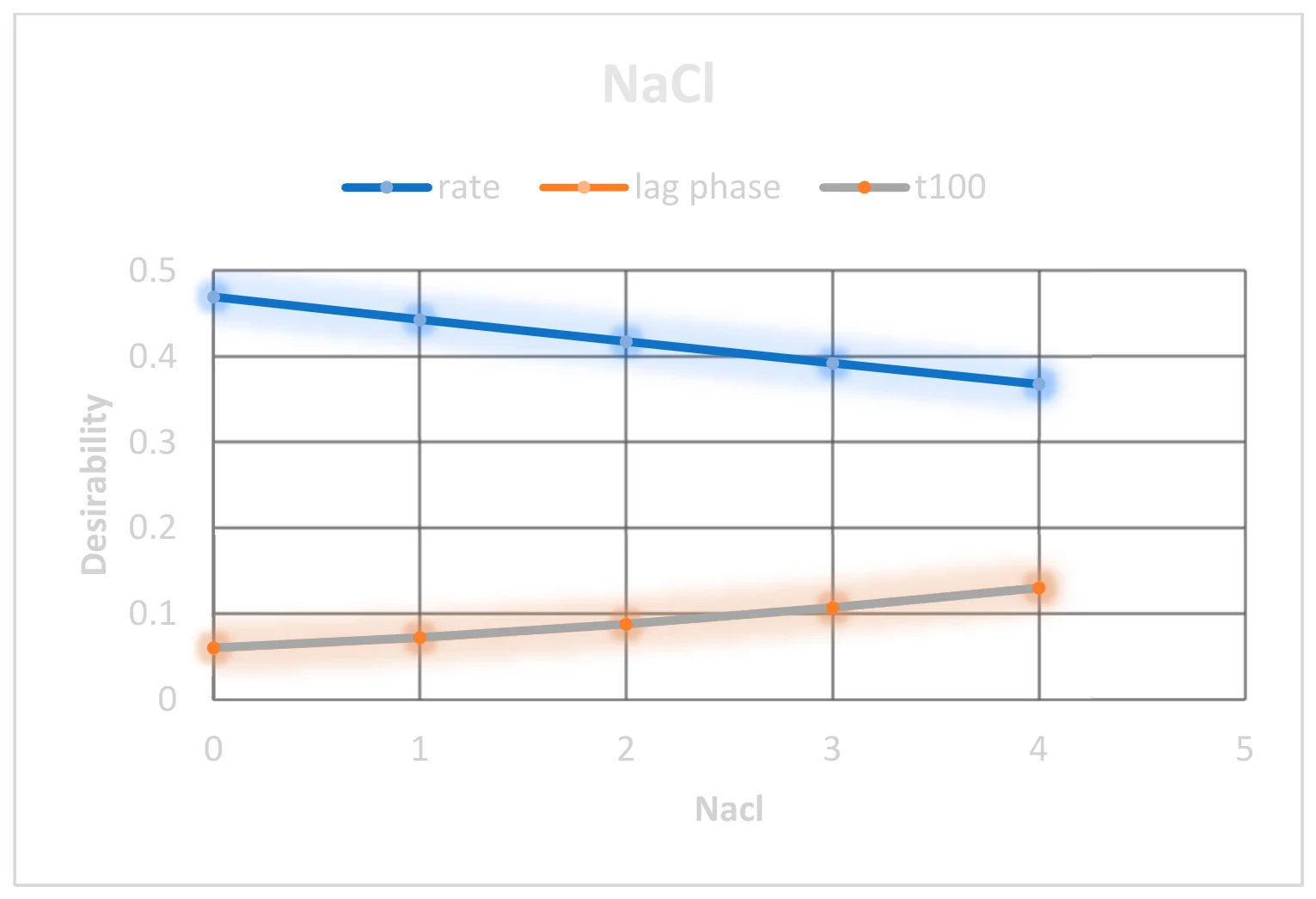
NaCl desirability profiles for kinetic parameters estimated with FSSP.

**Figure 4:**
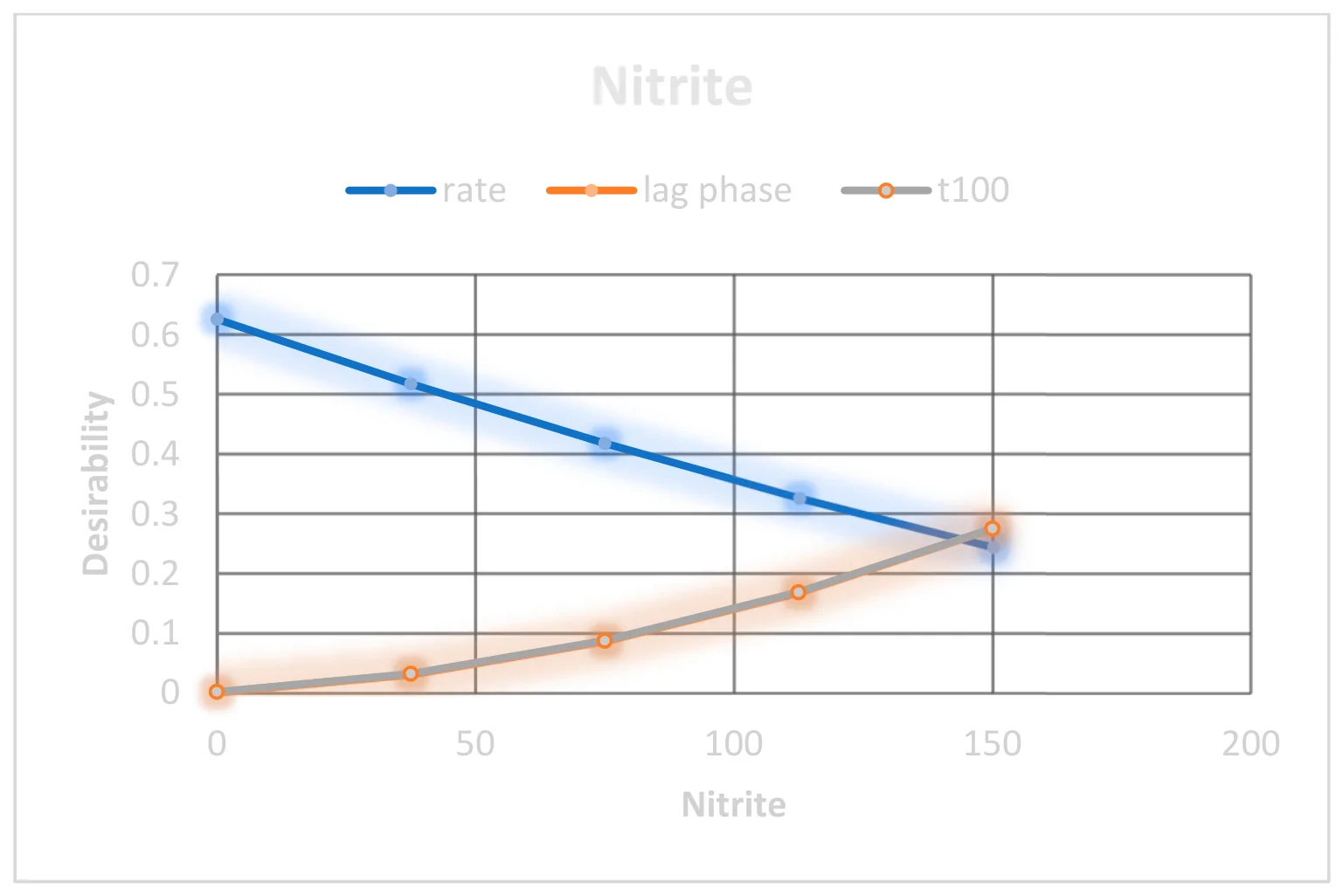
Nitrite desirability profiles for kinetic parameters estimated with FSSP.

**Figure 5:**
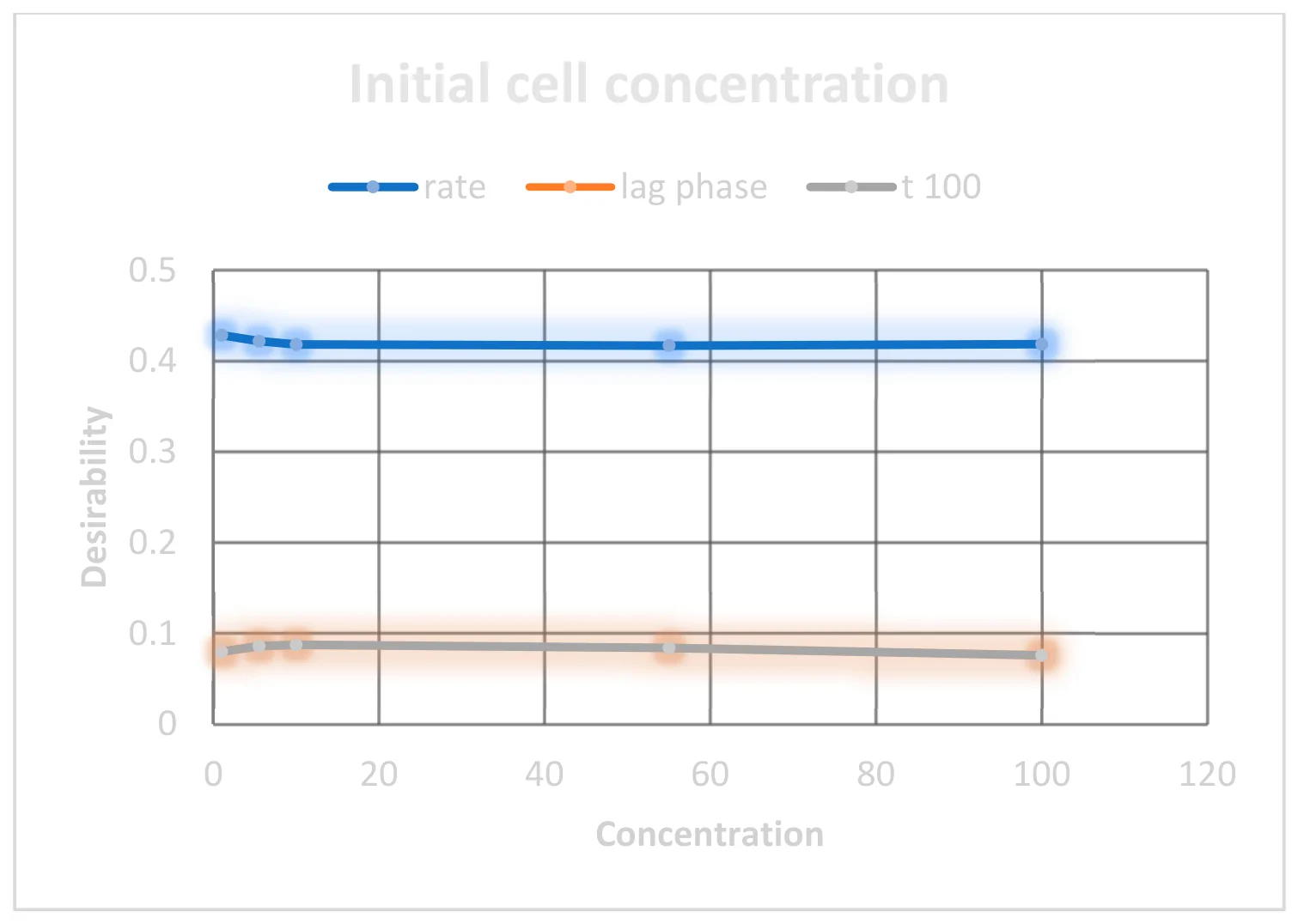
Initial cell concentration desirability profiles for kinetic parameters estimated with FSSP.

**Figure 6:**
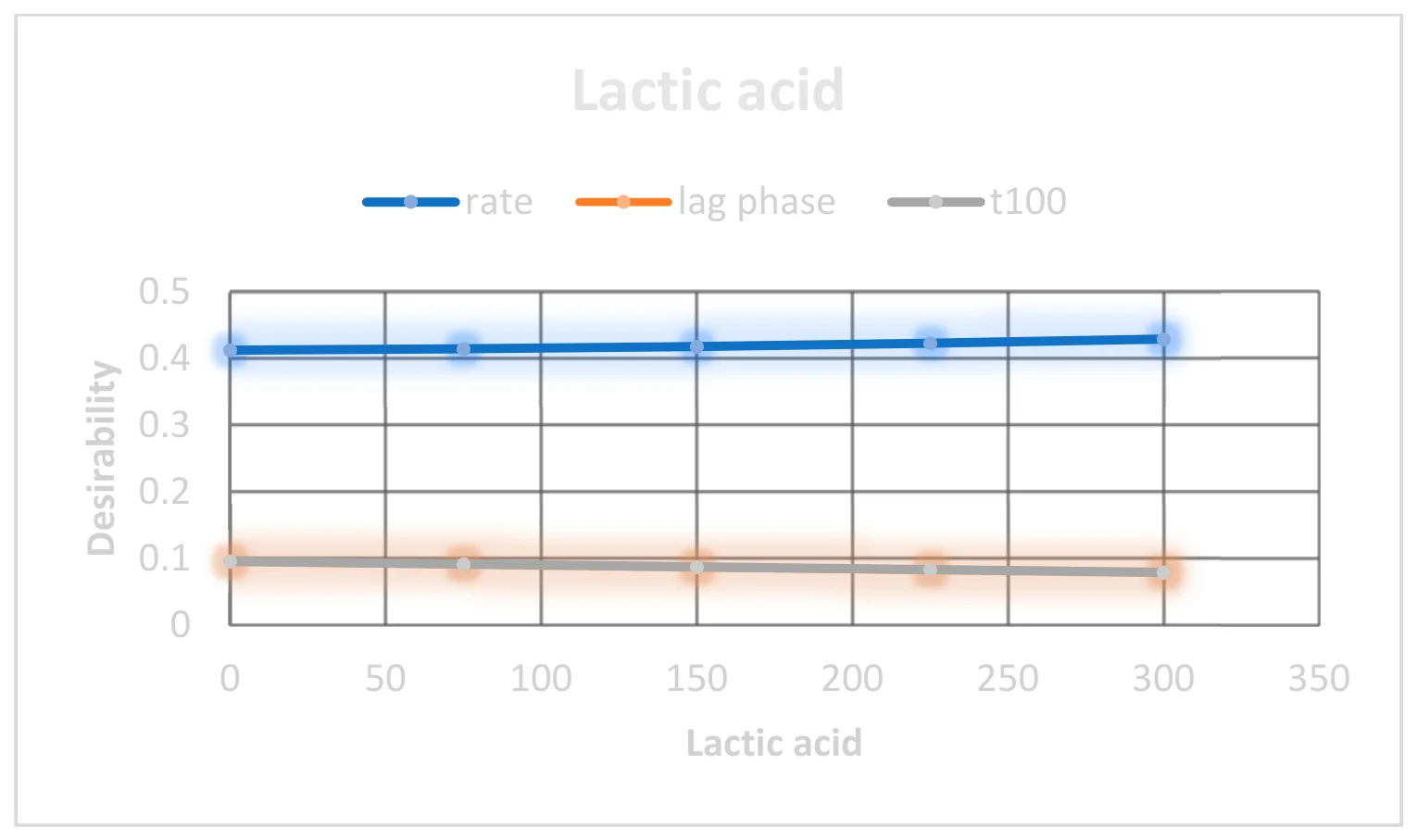
Lactic acid desirability profiles for kinetic parameters estimated with FSSP.

In contrast, sodium chloride exhibits an opposite trend compared to pH, confirming a marked inhibitory effect (Figure 3). Increasing salt concentration induces a progressive reduction in growth rate, accompanied by an extension of both the lag phase and *t*_100_. Sodium nitrite also showed a very clear dose-dependent inhibitory impact (Figure 4). At high nitrite concentrations, the desirability of the growth rate drops significantly, resulting in a drastic parallel extension of the lag phase and the *t*_100_ parameter. Finally, the initial cell concentration (Figure 5) and lactic acid (Figure 6) did not show significant effects or relevant changes on *L. monocytogenes* growth kinetics within the tested experimental range.

Overall, the observed responses were consistent with the known effects of environmental factors on *L. monocytogenes*. However, from a methodological perspective, the desirability profiles highlighted a close correspondence between the lag phase and *t*_100_ across the tested conditions, indicating that the adaptation period substantially contributes to the variability of the latter. At the same time, unlike the lag phase alone, *t*_100_ also incorporates the effect of growth rate on the time required to reach a predefined population threshold. Therefore, while lag phase and growth rate describe distinct portions of the growth curve, *t*_100_ summarizes the cumulative outcome of the entire growth process in a single biologically meaningful parameter.

### 3.2. Desirability Profiles from PMP Simulations

Based on the results obtained from the FSSP simulations, *t*_100_ was selected for further evaluation as a synthetic descriptor of growth behavior. Therefore, desirability profiling of the PMP dataset was performed directly on this parameter to assess its robustness across an independent simulation platform (Table 1). The desirability profile of *t*_100_ confirmed temperature as the factor exerting the strongest influence on the estimated time required to reach the target population, showing a clear threshold effect. In the thermal range between 10 °C and 25 °C, there is a drastic drop in the desirability value, which leads to a severe shortening of the growth time. Beyond 25 °C, the curve shows a stabilization plateau, where further thermal increases do not significantly modify the response.

Instead, pH showed a strictly progressive, linear, and additive trend. Each increase in pH leads to a constant change in desirability, confirming that a less acidic environment favors growth kinetics and reduces the time needed to reach the critical population. Finally, sodium nitrite shows a markedly nonlinear dose-dependent antimicrobial effect. Up to a concentration of 75 ppm (coded level 0), the increase in desirability for the risk time is moderate. Beyond this value, the antimicrobial action accelerates sharply, showing a critical threshold around 100–125 ppm, and then reaches the maximum inhibitory effect at higher concentrations.

### 3.3. Selection of t_100_ as an Integrated Kinetic Endpoint

The first step of the analysis was aimed at identifying a kinetic descriptor suitable for the subsequent development of predictive models, that is, to point out a single response variable able to summarize the combined contribution of adaptation and subsequent population increase under heterogeneous environmental conditions.

The FSSP and PMP dataset allowed different kinetic descriptors to be compared across combinations of temperature, pH, NaCl, nitrite, initial concentration, and lactic acid. From a methodological perspective, the desirability profiles highlighted a close correspondence between lag phase and *t*_100_, but the latter parameter also incorporated the contribution of the subsequent growth phase.

Therefore, while lag phase and growth rate describe different portions of the growth curve, *t*_100_ provides a single time-to-threshold endpoint reflecting their cumulative outcome. This characteristic was considered particularly useful for predictive modeling, because the response can be directly expressed as time and interpreted in relation to the persistence of environmental conditions before a predefined increase in pathogen concentration occurs.

### 3.4. Expansion and Harmonization of the Simulation Datasets

After the preliminary analysis discussed in the previous sections, the simulation dataset was expanded to increase the coverage of the environmental domain. The final database contained 428 simulated combinations generated using FSSP and PMP, including the original experimental-design combinations and 300 additional simulations designed to improve coverage of both intermediate and boundary regions of the input space (Tables S3 and S4).

First, the response variable was harmonized. FSSP reported *t*_100_ in days, whereas PMP returned the corresponding endpoint in hours. Therefore, all PMP *t*_100_ values were converted to days before model development. Second, the analysis of FSSP datasets pointed out that five simulations are right-censored conditions as *t*_100_ was probably > 90 days (maximum time input for the software). The PMP experimental design contained replicated central-point combinations, representing identical input conditions generally used in lab experiments to assess the casual variability. For modeling purposes, only one representative combination was retained for predictive modeling to prevent the central point from receiving disproportionate statistical weight.

The environmental domains represented by the two simulation sources were not completely identical. In particular, the FSSP dataset included a wider temperature range and lower initial inoculum levels, whereas PMP extended the range of initial concentrations towards substantially higher values. For this reason, simulation source was initially retained as a candidate.

Finally, initial concentration was log10-transformed before model development because of its wide numerical range. The common predictors used in the integrated analysis were therefore temperature, pH, NaCl concentration, nitrite concentration, log10 initial concentration, and simulation source. Lactic acid was used for a second modeling approach using the dataset from FSSP.

### 3.5. Comparison of Different Models

Four approaches of increasing complexity were compared: a nonlinear logistic model based exclusively on environmental predictors, a nonlinear logistic model including simulation source, a logistic formulation allowing interactions between source and environmental factors, and Random Forest regression. In addition to model fitting performance, predictive performance was assessed by an internal 5-fold cross-validation.

The basic logistic model provided a training R^2^ of 0.373 and a cross-validated R^2^ of only 0.028, with a cross-validated RMSE of 8.07 days. Including simulation source improved the model moderately, increasing training R^2^ to 0.446 and cross-validated R^2^ to 0.133, while decreasing cross-validated RMSE to 7.63 days (Table 2).

Allowing source-dependent interactions with environmental variables produced a further improvement. The resulting model achieved a training R^2^ of 0.637 and a cross-validated R^2^ of 0.344, with an RMSE of 6.64 days. The improvement obtained after introducing source × environmental-factor interactions indicates that the relationship between environmental conditions and *t*_100_ was not completely invariant between FSSP and PMP.

On the other hand, Random Forest showed a better predictive performance, with a training R^2^ close to 0.97 and a cross-validated R^2^ of approximately 0.70, with an RMSE of 4.5 days. Based on these results, Random Forest was selected as suitable model.

### 3.6. Contribution of the Simulation Source and Weight of Lactic Acid in the Random Forest

The preliminary evaluation of the regression models showed that the inclusion of the simulation source (FSSP or PMP) might improve the performance of the model, thus suggesting that systematic differences between FSSP and PMP had to be explicitly considered. Therefore, the source was included as an input variable and the statistical weight of the different variables was assessed, as reported in Table 3. Temperature (38%) and NaCl (38.8%) were the dominant predictors, followed by nitrite (15.5%) and pH (6.1%). In contrast, simulation source accounted for limited permutation importance, along with the initial concentration (<1%).

This result should not be interpreted as demonstrating equivalence between FSSP and PMP. The two platforms differ in their underlying datasets, model formulations, and represented environmental domains. Rather, the relatively low permutation importance of simulation source indicates that, after the environmental predictors and their nonlinear relationships were accounted for by the Random Forest, source identity provided only a limited additional contribution within the represented simulation domain.

After assessing the “weight” of the source, the effect of lactic acid was evaluated. It represented a methodological issue because it was available as an input variable in FSSP but not in PMP. Therefore, it could not be included directly in the integrated FSSP–PMP model without introducing a structural confounding effect. Therefore, an additional sensitivity analysis was conducted using only FSSP simulations. Applying 5-fold cross-validation did not show an improvement, as the model without lactic acid achieved an average R^2^ of 0.657 ± 0.249 and an RMSE of 4.92 ± 1.32 days, while with lactic acid the corresponding values were R^2^ = 0.631 ± 0.262 and RMSE = 5.12 ± 1.38 days.

Permutation importance supported the same conclusion. In the FSSP model including all six predictors, NaCl accounted for approximately 42.6% of normalized permutation importance, temperature for 39.0%, and nitrite for 16.4%. The contribution of pH was approximately 1.1%, whereas lactic acid and initial concentration accounted for only approximately 0.48% and 0.43%, respectively (Table 4).

As a result of this modeling step, lactic acid was not included in the final model.

### 3.7. Hyperparameter Optimization of the Random Forest and Variable Importance

After Random Forest selection as the most suitable model for *t*_100_, hyperparameter optimization was performed to reduce unnecessary model complexity and identify a more robust configuration. The process considered the following variables: tree depth, proportion of predictors evaluated at each split, minimum leaf size, and minimum number of observations required to split an internal node. Model configurations were compared according to cross-validated RMSE rather than training performance.

The selected structure was characterized by a maximum tree depth of 8, max_features = 0.85, min_samples_leaf = 1, and min_samples_split = 3. After selection of the structural hyperparameters, the final model was fitted using 1000 trees to stabilize ensemble predictions.

The optimized Random Forest achieved a mean training R^2^ of 0.942, with a corresponding RMSE of 1.96 days. More importantly, repeated 5-fold cross-validation yielded: R^2^ = 0.724 ± 0.132 and RMSE = 4.05 ± 1.42 days.

The out-of-bag R^2^ of the final model was 0.759, providing an additional internal estimate broadly consistent with the repeated cross-validation results.

A separate single 5-fold cross-validation, used to obtain one cross-validated prediction for each observation for diagnostic plotting, resulted in an overall R^2^ of 0.700 and RMSE of 4.50 days.

Permutation importance calculated for the optimized model confirmed the hierarchy observed during preliminary Random Forest analysis. Temperature represented the most influential predictor, accounting for 39.67% of normalized permutation importance, followed by NaCl at 38.03%. Nitrite contributed 14.58%, while pH accounted for approximately 6.01%; the contributions of simulation source and initial inoculum were very small, corresponding to 1.00% and 0.72%, respectively (Table 5). The stability of this ranking before and after hyperparameter optimization indicates that the identified hierarchy was not dependent on a specific Random Forest configuration.

### 3.8. Prediction Error of Random Forest Across *t*_100_ Domain

The performance metrics (R^2^, RMSE, for both model training and internal validation) provide an average measure of accuracy; however, they cannot show if the fitting performances are homogeneous across the response domain. For this purpose, the observations were divided into quartiles according to *t*_100_ values. Prediction performance was highest for intermediate and relatively short *t*_100_ values; for example, for the second quartile (*t*_100_ values between 1.13 and 2.61 days), RMSE was only 1.52 days and MAE 0.87 days. In contrast, the error increased in the upper quartile (*t*_100_ values between 5.51 and 62.90 days); in this region, RMSE increased to 7.45 days and MAE to 4.49 days (Table 6). The positive mean residual in the upper quartile indicates that the model tended to predict lower values than those observed when *t*_100_ became particularly long.

### 3.9. Applicability Domain

To further investigate whether model performance was affected by the position of each simulated condition within the environmental space, an applicability-domain analysis was performed. Each of the combinations included in the regression analysis was classified as either an interior- or a boundary-domain observation according exclusively to the values of the input variables. The 10th and 90th percentiles were calculated for each of the five environmental predictors included in the integrated model: temperature, pH, NaCl concentration, nitrite concentration, and initial cell concentration. A combination was classified as a boundary-domain observation when at least one of the five predictors was equal to or below its source-specific 10th percentile or equal to or above its source-specific 90th percentile. On the other hand, combinations for which all five predictors were between their respective source-specific 10th and 90th percentiles were classified as interior-domain observations. Table 7 shows the percentiles.

Following this method, 194 interior-domain and 224 boundary-domain combinations were identified. A marked difference in predictive accuracy was observed between the two regions. For the 194 interior-domain combinations, RMSE was 1.26 days and MAE 0.84 days, whereas the 224 boundary-domain combinations showed an RMSE of 6.03 days and an MAE of 3.09 days. Mean residuals were close to zero in both groups (−0.07 and +0.12 days for interior and boundary observations, respectively) (Table 8). These results suggest that the reduction in predictive performance at the boundaries was mainly associated with an increased dispersion of prediction errors rather than with a systematic tendency towards over- or underprediction. As a consequence, the optimized Random Forest was accurate when interpolating among conditions located within the central portion of the represented environmental space, whereas prediction uncertainty increased when one or more environmental variables approached the extremes of the source-specific simulation domain.

### 3.10. Final Random Forest Model and Graphical Representation

After all steps the optimized Random Forest was retained as the most suitable model for *t*_100_ of *L. monocytogenes*. The model used temperature, pH, NaCl concentration, nitrite concentration, log_10_-transformed initial cell concentration, and simulation source (FSSP or PMP) as independent variable. The final ensemble consisted of 1000 regression trees, with a maximum tree depth of 8, max_features = 0.85, min_samples_leaf = 1, and min_samples_split = 3.

Unlike parametric regression models, Random Forest does not provide a single equation containing fitted coefficients. Its prediction can instead be formally expressed as the average prediction generated by the individual regression trees, and the general formula is:

x = [T, pH, NaCl, nitrite, log_10_(N_0_), source]

$${\hat{t}}_{100}^{\left(i\right)}=\frac{1}{N}\sum_{k=1}^{N}h_{k}(X_{i})$$

**Figure 7:**
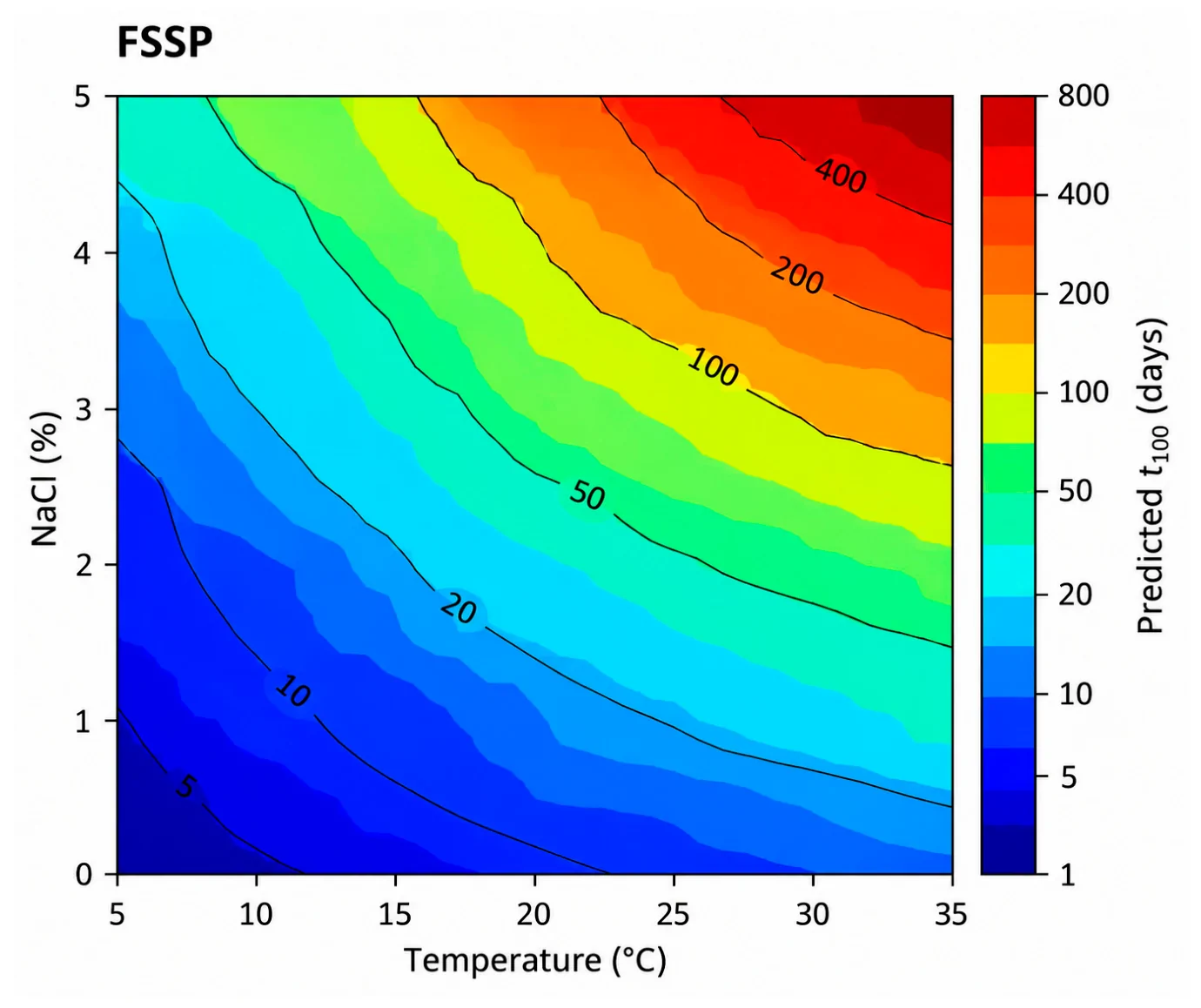
Response surface of the optimized Random Forest model showing the predicted *t*_100_ of *L. monocytogenes* as a function of temperature and NaCl concentration for FSSP simulations. The remaining predictors were fixed at representative values (pH = 6.5, nitrite = 75 ppm, initial cell concentration = 50 CFU/g), while source was set to FSSP.

To provide a graphical representation, predicted *t*_100_ values were used to generate two-dimensional response surfaces. Considering the relative importance of the predictors, the interactions temperature × NaCl were built (Figure 7). The response surfaces showed a marked increase in predicted *t*_100_ under combinations of conditions progressively less favorable to *L. monocytogenes* growth (decreased temperature and increased salt concentration). The surfaces also highlighted the nonlinear nature of the relationship.

**Figure 8:**
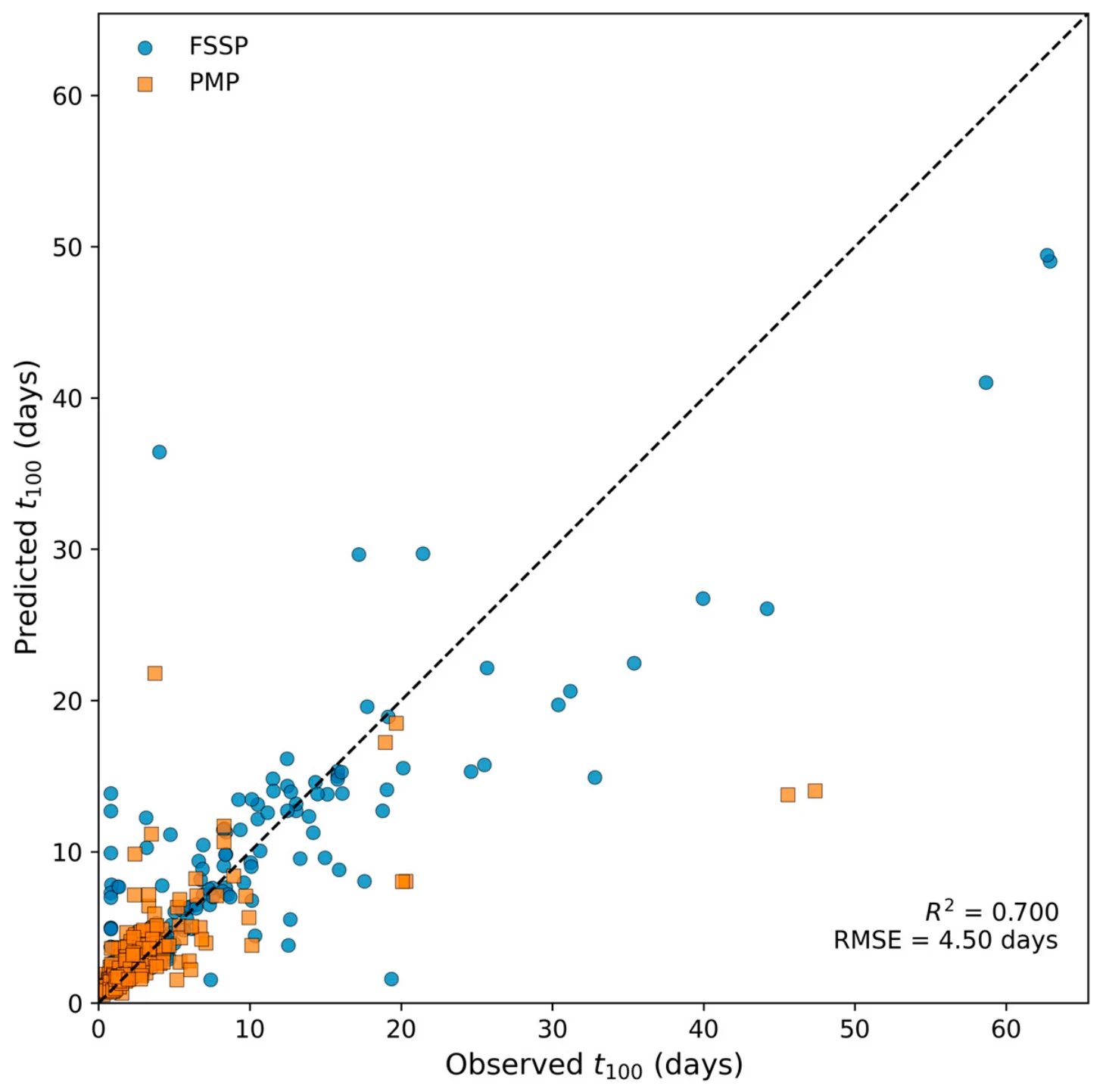
Observed versus predicted *t*_100_ values obtained with the optimized Random Forest model using 5-fold cross-validation. FSSP and PMP simulations are shown separately. RMSE, root mean squared error.

The agreement between observed and cross-validated predictions and the corresponding residual distribution were examined graphically (Figure 8). The observed-versus-predicted plot showed an overall agreement between predicted and simulated *t*_100_. Greater dispersion was observed at higher predicted values, as also reported in Section 3.8 and Section 3.9, which showed increasing prediction error for long *t*_100_ values and for combinations located near the boundaries of the represented environmental domain.

## 4. Discussion

The main objective of this study was to identify a kinetic endpoint able of integrating different components of *L. monocytogenes* growth dynamics and to evaluate whether this endpoint could subsequently be modeled using data generated by two different predictive microbiology platforms. The results support the use of *t*_100_ as a useful time-to-threshold descriptor. The close similarity between the desirability profiles of lag phase and *t*_100_ observed in the preliminary analysis indicates that adaptation time strongly contributes to this endpoint, although *t*_100_ also incorporates the contribution of the subsequent growth rate. This result confirms the importance of lag-phase dynamics in predictive microbiology in general and in particular for *L. monocytogenes* [20,21,22]. In this context, *t*_100_ should not be regarded as a new fundamental kinetic parameter, but rather as an operational endpoint combining information that would otherwise be distributed among different kinetic descriptors.

The inclusion of the lag phase in the calculation of *t*_100_ is particularly relevant when microorganisms are exposed to suboptimal environmental conditions. Under such conditions, cells may require a period of physiological adaptation before active multiplication occurs, and estimates based exclusively on exponential growth rates may therefore provide an incomplete representation of the time required to reach a critical population level.

A major methodological issue in the present study was the integration of simulations generated by FSSP and PMP. Predictive microbiology tools may differ in their underlying datasets, mathematical formulations, assumptions, and applicability domains, and discrepancies among predictive systems may therefore contribute to model uncertainty [23,24]. Consequently, integration of FSSP and PMP outputs was not based on an assumption of equivalence between the two platforms. Instead, simulation source was explicitly incorporated into the modeling strategy, and models of increasing complexity were evaluated to determine whether a common predictive framework could accommodate both shared environmental effects and source-dependent differences. The improvement observed when source was included in the nonlinear logistic model, and the further improvement obtained by introducing source × environmental-factor interactions, indicated that the relationships between environmental conditions and *t*_100_ were not completely invariant between FSSP and PMP.

The expansion of the preliminary dataset was also important for evaluating more flexible predictive approaches. The original simulation space was supplemented with 300 additional combinations, equally distributed between FSSP and PMP, thus increasing the number and heterogeneity of conditions available for model development. Among the modeling approaches evaluated, Random Forest provided the best predictive performance. This algorithm is particularly suitable for identifying nonlinear relationships and interactions without requiring a predefined functional relationship between predictors and response [25]. Following hyperparameter optimization, repeated 5-fold internal cross-validation yielded an average R^2^ of 0.724 ± 0.132 and an RMSE of 4.05 ± 1.42 days. The out-of-bag R^2^ of 0.759 provided an additional internal estimate broadly consistent with these results, while the separate single 5-fold cross-validation used to generate one prediction for each observation yielded R^2^ = 0.700 and an RMSE of 4.50 days. Importantly, all these statistics represent estimates of internal predictive performance and should not be interpreted as independent external validation.

Permutation importance provided additional insight into the structure of the optimized Random Forest. Temperature and NaCl were the dominant predictors, accounting for approximately 39.7% and 38.0% of normalized permutation importance, respectively, followed by nitrite (14.6%) and pH (6.0%). These findings are consistent with the known importance of environmental hurdles in determining the growth kinetics of *L. monocytogenes* [20,22]. Conversely, simulation source and initial inoculum accounted for only approximately 1.0% and 0.7%, respectively. The low permutation importance of simulation source should not be interpreted as demonstrating equivalence between FSSP and PMP. The two platforms differ in their structures and represented domains [24]. Rather, the result indicates that, after the environmental predictors and their nonlinear relationships were accounted for by the Random Forest, source identity provided a relatively small additional contribution to prediction within the represented simulation dataset.

Lactic acid represented a methodological issue because it is available as an input variable in FSSP but not in PMP. Direct inclusion of this variable in the integrated dataset would therefore have introduced structural confounding between lactic acid availability and simulation source. Its contribution was evaluated in a separate FSSP-specific sensitivity analysis. Inclusion of lactic acid did not improve cross-validated Random Forest performance. These findings should not be interpreted as evidence that lactic acid is biologically irrelevant to *L. monocytogenes*. They indicate only that, within the combinations represented in the present FSSP simulation dataset, lactic acid provided limited additional predictive information relative to the dominant environmental predictors. Its exclusion from the integrated model is therefore supported both by its source-specific availability and by the results of this sensitivity analysis.

The distribution of prediction errors showed that model performance was not homogeneous across the *t*_100_ range. Errors were relatively small for short and intermediate *t*_100_ values but increased in the upper quartile of the response distribution.

A consistent pattern emerged from the applicability-domain analysis. Conditions located within the central, non-extreme portion of the source-specific environmental space showed substantially lower prediction errors than combinations in which at least one environmental predictor approached the empirical extremes of its distribution, reflecting an increased dispersion of prediction errors rather than a systematic bias. These results emphasize that model predictions should be interpreted in relation to the environmental space represented in the training data and that increased caution is required when predictor values approach the boundaries of that space.

Several limitations should be considered when interpreting this model. Most importantly, all observations used for its development originated from FSSP and PMP simulations. Cross-validation, repeated cross-validation and out-of-bag estimation quantify internal predictive performance within this simulated dataset, but none constitutes independent experimental validation. The model inevitably inherits assumptions and potential biases from the source predictive systems [23]. Moreover, real foods introduce structural and compositional complexity that cannot be fully represented by the environmental predictors considered here. Food microstructure can influence microbial growth through spatial constraints and heterogeneous distributions of nutrients and inhibitory compounds [26]. In addition, strain-to-strain variability in *L. monocytogenes* may affect growth and stress responses [27], while interactions with the resident microbiota, including lactic acid bacteria, may further modify pathogen behavior [28]. Consequently, the predictive performance reported in this study should not be directly extrapolated to real food matrices.

Independent experimental validation therefore represents the essential next step. Challenge tests performed in controlled systems and, more importantly, in representative food matrices would allow model predictions to be compared with observations independent of both FSSP and PMP. Such experiments would provide estimates of prediction bias and accuracy under actual food conditions and establish whether recalibration or matrix-specific correction factors are required. Future extensions could also consider strain-level variability; the increasing integration of genomic information into quantitative microbial risk assessment provides one possible strategy for incorporating this additional source of biological heterogeneity [27,29].

For the same reason, the present results do not support direct use of the model for regulatory compliance or process validation. Such applications require evidence that predictions remain reliable when confronted with independent empirical observations under relevant food-processing and storage conditions. Experimental validation is therefore required before the applicability of the proposed framework to food-safety decision making or process-validation contexts can be assessed. Similarly, integration with real-time environmental measurements, IoT sensors, cloud platforms or other digital decision-support systems should be regarded as a possible future development rather than as an outcome of the present study. Such technological implementation should only be considered following successful independent validation.

The principal contribution of this study is methodological. The results support *t*_100_ as an integrated time-to-threshold endpoint, demonstrate a strategy for combining heterogeneous outputs from FSSP and PMP while explicitly accounting for simulation source, and show that an optimized Random Forest can reproduce the resulting multidimensional response space with substantially better internal predictive performance than the parametric models evaluated. At the same time, the error distribution, applicability-domain analysis and behavior under strongly growth-limiting conditions define important boundaries for model interpretation.

## 5. Conclusions

This study supports the use of *t*_100_, defined as the time for a 2-log increase in *L. monocytogenes* population, as an integrated time-to-threshold endpoint capable of combining information related to microbial adaptation and subsequent growth, allowing the combination of inputs from two sources (FSSP and PMP) into a common modeling framework. The optimized Random Forest provided the best internal predictive performance, with repeated 5-fold cross/internal-validation yielding an average R^2^ of 0.724 ± 0.132 and an RMSE of 4.05 ± 1.42 days. Temperature and NaCl were the dominant predictors, while the simulation source had a small predictive contribution. Similarly, the FSSP-specific analysis indicated that inclusion of lactic acid did not improve model performance within the investigated simulation domain. The simulation analysis also identified some limits, as prediction error increases for high values of *t*_100_ or near the boundaries of the represented environmental space; most importantly, the model was designed and developed from simulated data, thus an external validation and some confirmatory challenge tests are required for assessing possible applications in real scenarios.

## Figures and Tables

**Table 1:** Desirability profiles for temperature, pH, and sodium nitrite at *t*_100_, estimated using PMP parameters.

Coded Levels	Temperature	pH	Nitrite
−2	0.574	0.541	0.080
−1	0.337	0.321	0.129
0	0.181	0.181	0.181
1	0.106	0.121	0.238
2	0.112	0.140	0.298

**Table 2:** Performance of the candidate models during model fitting and internal 5-fold cross-validation. In each cross-validation iteration, approximately 80% of the observations were used for model fitting and the remaining 20% for validation. RMSE, root mean squared error; R^2^, coefficient of determination.

Model	Training-R^2^	5-Fold CV R^2^	RMSE (Days)
Nonlinear logistic	0.373	0.028	8.07
Nonlinear logistic + Source	0.446	0.133	7.63
Logistic + Source interactions	0.637	0.344	6.64
Random Forest	0.97	0.70	4.5

**Table 3:** Permutation important of the different variables in the Random Forest analysis.

Variable	Permutation Importance
NaCl	38.8%
Temperature	38.0%
Nitrite	15.5%
pH	6.1%
Source	0.9%
Initial concentration	0.7%

**Table 4:** Permutation importance for the FSSP-specific Random Forest including lactic acid.

Variable	Permutation Importance
NaCl	42.6%
Temperature	39.0%
Nitrite	16.4%
pH	1.1%
Lactic acid	0.48%
Initial concentration	0.43%

**Table 5:** Normalized permutation importance of the predictors in the optimized integrated Random Forest.

Variable	Permutation Importance
Temperature	39.67%
NaCl	38.03%
Nitrite	14.58%
pH	6.01%
Simulation source	1.00%
Initial concentration	0.72%

**Table 6:** Prediction error according to the observed *t*_100_ domain. Random Forest model. RMSE, root mean square error; MAE, mean absolute error.

*t*_100_ Interval	n	RMSE (Days)	MAE (Days)	Mean Residual (Days)
0.02–1.13	105	2.48	1.20	−1.19
1.13–2.61	104	1.52	0.87	−0.70
2.62–5.42	104	4.10	1.61	−0.88
5.51–62.90	105	7.45	4.49	+2.89

**Table 7:** Source-specific thresholds used to define the applicability domain and distinguish between interior and boundary combinations.

	FSSP P10	FSSP P90	PMP P10	PMP P90
Temperature (°C)	7.70	35.00	10.01	28.09
pH	5.70	7.70	5.091	6.877
NaCl	0.00	4.495	1.164	4.787
Nitrite (ppm)	0.00	150.00	6.86	131.87
Initial concentration (CFU/g)	1.00	100.00	18.33	45,563

**Table 8:** Predictive performance of the optimized Random Forest model in the interior and boundary regions of the simulation domain. Interior: All five predictor values were >P10 and <P90 within the corresponding simulation source. Boundary: At least one predictor was ≤P10 or ≥P90 within the corresponding simulation source.

Domain	n	RMSE (Days)	MAE (Days)	Mean Residual (Days)
Interior	194	1.26	0.84	−0.07
Boundary	224	6.03	3.09	+0.12

## Data Availability

The original contributions presented in this study are included in the article/ Supplementary Materials. Further inquiries can be directed to the corresponding author.

## Supplementary Materials

The following supporting information can be downloaded at https://www.mdpi.com/article/10.3390/foods15203640/s1, Table S1: Simulations with FSSP. Data used for DoE analysis; Table S2: Simulations with PMP. Data used for DoE analysis; Table S3: Additional combinations of FSSP; Table S4: Additional combinations of PMP.
