# Supplementary material for: A Machine Learning Approach to Predict Listeria monocytogenes Growth by Integrating FSSP and PMP Simulations: A Focus on t100 as a Possible Endpoint Parameters for Predictive Models
Source: Foods. 2026 Oct 14;15(20):3640. doi: 10.3390/foods15203640 (PMC13704739; doi:10.3390/foods15203640)
Supplement: Supplementary file 1 [file foods-15-03640-s001.zip › foods-4505327-supplementary.pdf]

Table S1. Simulations with FSSP. Data used for DoE analysis

Initial concentration (CFU/g)	NaCl (%)	Nitrite (ppm)	Lactic acid (ppm)	pH	Temperature (°C)	Growth rate (CFU/h)	Lag phase (d)	t_{100} (d)
1	2	0	0	5.7	35	0.3617	0.32	0.8
1	0	0	150	7.7	25	0.3043	0.32	0.8
100	0	0	150	5.7	25	0.2707	0.32	0.8
1	0	0	0	5.7	15	0.1634	1.0338	2.5665
10	0	150	150	7.7	15	0.0521	2.3679	5.8665
10	0	75	300	7.7	25	0.2344	0.3396	0.8427
10	0	0	300	5.7	25	0.2757	0.32	0.8
100	2	150	0	7.7	15	0.0478	2.72	6.74
10	2	0	150	6.7	35	0.3774	0.32	0.8
10	2	75	0	6.7	25	0.1487	0.87	2.16
100	0	0	150	5.7	35	0.3792	0.32	0.8
100	2	150	150	6.7	25	0.1144	1.14	2.81
1	4	75	300	5.7	15	0.0789	1.65	4.08
100	4	150	300	5.7	25	0.0767	1.69	4.19
10	2	75	300	7.7	15	0.0727	1.79	4.42
100	4	0	0	6.7	35	0.3538	0.32	0.8
100	0	75	150	7.7	35	0.3378	0.32	0.8
1	4	150	0	6.7	25	0.0937	1.39	3.43
100	0	0	300	7.7	15	0.1947	0.6931	1.7205
100	4	75	0	5.7	35	0.2682	0.32	0.8
100	4	75	300	6.7	25	0.1769	0.73	1.82
100	2	75	150	7.7	25	0.22	0.59	1.46
10	0	75	0	6.7	35	0.3222	0.32	0.8
10	2	0	0	7.7	25	0.3564	0.36	0.9
1	0	150	0	6.7	15	0.04	2.5382	6.2896
1	0	75	300	5.7	35	0.3154	0.32	0.8
10	4	150	0	5.7	15	0.04	4.07	10.09
100	2	150	300	5.7	35	0.2219	0.4092	1.0129
10	4	0	300	5.7	35	0.3471	0.32	0.8
100	2	75	300	6.7	35	0.3075	0.32	0.8

1	4	0	150	7.7	35	0.3756	0.32	0.8
10	2	75	150	5.7	35	0.2918	0.32	0.8
1	0	75	0	7.7	15	0.122	1.6195	4.015
1	4	150	150	5.7	35	0.2044	0.7101	1.7571
10	4	0	0	7.7	15	0.1198	1.08	2.69
1	2	150	300	7.7	25	0.1164	1.12	2.77
1	2	0	150	7.7	15	0.1463	0.89	2.2
10	4	75	300	7.7	35	0.3057	0.32	0.8
1	0	0	300	6.7	35	0.401	0.32	0.8
1	0	150	150	5.7	25	0.1331	1.4287	3.5403
100	0	150	300	5.7	15	0.04	2.5306	6.2696
10	0	150	300	6.7	25	0.1488	1.2583	3.1173
100	4	150	150	6.7	15	0.04	3.38	8.37
10	2	0	300	5.7	15	0.1178	1.1	2.73
10	0	150	0	5.7	35	0.2365	0.32	0.8
100	0	75	0	5.7	25	0.1969	0.6398	1.5895
10	2	150	150	7.7	35	0.2505	0.32	0.8
100	4	75	150	7.7	15	0.074	1.76	4.35
1	2	75	300	5.7	25	0.1772	0.73	1.82
10	0	75	150	5.7	15	0.0934	1.7822	4.4181
100	4	0	300	7.7	25	0.2918	0.45	1.1
1	2	150	150	5.7	15	0.04	3.36	8.31
1	0	150	300	7.7	35	0.2741	0.32	0.8
1	4	150	300	7.7	15	0.04	3.32	8.23
1	4	0	300	6.7	15	0.1176	1.1	2.73
100	2	0	0	6.7	15	0.1438	0.9	2.24
1	2	0	300	6.7	25	0.35	0.37	0.92
100	2	0	300	7.7	35	0.3931	0.32	0.8
10	4	75	0	6.7	15	0.0727	1.79	4.42
100	4	0	150	5.7	15	0.0971	1.34	3.32
10	2	150	300	6.7	15	0.0469	2.77	6.86
10	4	150	150	7.7	25	0.0953	1.36	3.38
100	0	75	300	6.7	15	0.1091	1.6119	3.995

100	0	150	150	6.7	35	0.2522	0.32	0.8
10	2	150	0	5.7	25	0.0777	1.67	4.14
1	2	150	0	6.7	35	0.2348	0.4168	1.0329
100	0	150	0	7.7	25	0.1555	1.2255	3.0379
1	0	75	150	6.7	25	0.2187	0.5099	1.2658
1	2	75	0	7.7	35	0.3204	0.32	0.8
10	4	0	150	6.7	25	0.2868	0.45	1.12
10	0	0	150	6.7	15	0.179	0.8635	2.1435
1	2	75	150	6.7	15	0.0887	1.46	3.63
100	0	0	0	6.7	25	0.2825	0.32	0.8
10	4	150	300	6.7	35	0.2201	0.5397	1.334
1	4	0	0	5.7	25	0.238	0.55	1.35
100	4	150	0	7.7	35	0.2268	0.507	1.2546
10	0	0	0	7.7	35	0.4078	0.32	0.8
1	4	75	150	6.7	35	0.29	0.32	0.8
10	4	75	150	5.7	25	0.146	0.89	2.2
1	4	75	0	7.7	25	0.1802	0.72	1.79
100	2	75	0	5.7	15	0.0736	1.76	4.37

Table S2. Simulations with PMP. Data used for DoE analysis

Temperature (°C)	pH	NaCl (%)	Nitrite (ppm)	Initial concentration (Log CFU/g)	Maximal cell concentration (Log CFU/g)	Growth rate ((Log CFU/g)/h)	Lag phase (h)	t_{100} (h)
15	5.5	2	35	2	7.324	0.065	27.967	102.804
15	5.5	2	35	4	5.340	0.065	28.026	69.996
15	5.5	2	105	2	7.195	0.042	45.319	163.500
15	5.5	2	105	4	5.286	0.041	45.248	111.562
15	5.5	4	35	2	4.642	44.530	26.764	55.261
15	5.5	4	35	4	2.663	9.730	62.017	85.379
15	5.5	4	105	2	4.667	19.670	52.235	128.500
15	5.5	4	105	4	2.353	1.348	237.160	242.869
15	6.5	2	35	2	7.327	0.062	23.056	102.256
15	6.5	2	35	4	5.343	0.061	23.128	67.456
15	6.5	2	105	2	7.354	0.090	15.903	70.149
15	6.5	2	105	4	5.351	0.090	16.044	46.339
15	6.5	4	35	2	7.348	0.112	18.356	62.087
15	6.5	4	35	4	5.345	0.111	18.425	42.829
15	6.5	4	105	2	7.342	0.086	23.885	80.550
15	6.5	4	105	4	5.343	0.086	23.937	55.554
25	5.5	2	35	2	7.372	0.136	10.644	46.902
25	5.5	2	35	4	5.359	0.135	10.828	31.081
25	5.5	2	105	2	7.354	0.086	17.611	74.818
25	5.5	2	105	4	5.354	0.085	17.795	49.838
25	5.5	4	35	2	7.359	0.121	16.697	57.275
25	5.5	4	35	4	5.351	0.120	16.843	39.574
25	5.5	4	105	2	7.333	0.076	27.307	91.894
25	5.5	4	105	4	5.344	0.075	27.399	63.754
25	6.5	2	35	2	7.380	0.278	4.483	22.175
25	6.5	2	35	4	2.641	0.195	14.471	4.173

25	6.5	2	105	2	7.372	0.215	5.998	28.812
25	6.5	2	105	4	5.355	0.213	4.747	17.506
25	6.5	4	35	2	7.354	0.270	7.053	25.179
25	6.5	4	35	4	5.347	0.269	7.098	17.200
25	6.5	4	105	2	7.351	0.207	9.301	32.852
25	6.5	4	105	4	5.346	0.207	9.343	22.462
10	6.0	3	70	3	6.260	0.040	51.889	147.176
30	6.0	3	70	3	6.364	0.197	7.729	26.952
20	5.0	3	70	3	6.138	0.040	50.394	145.939
20	7.0	3	70	3	6.366	0.194	7.453	26.982
20	6.0	1	70	3	6.350	0.142	9.880	36.499
20	6.0	5	70	3	6.356	0.115	21.158	54.027
20	6.0	3	0	3	6.368	0.165	9.749	32.720
20	6.0	3	140	3	6.348	0.081	21.220	67.880
20	6.0	3	70	1	8.360	0.117	14.542	66.442
20	6.0	3	70	5	4.347	0.115	14.796	29.536
20	6.0	3	70	3	6.355	0.116	14.668	47.253

Table S3. Additional combination of FSSP.

Initial concentration (CFU/g)	NaCl (%)	Nitrite (ppm)	Lactic acid (ppm)	pH	Temperature (°C)	t_{100} (d)
1.00	1.00	150.00	300.00	7.70	5.00	32.81
100.00	5.00	0.00	0.00	5.70	25.00	19.36
1.00	1.00	0.00	0.00	7.70	25.00	1.02
1.00	5.00	0.00	300.00	5.70	5.00	15.79
100.00	1.00	150.00	0.00	5.70	5.00	0
100.00	5.00	150.00	300.00	7.70	25.00	3.83
1.00	1.00	0.00	300.00	5.70	25.00	1.03
1.00	1.00	150.00	0.00	5.70	25.00	3.13
1.00	5.00	0.00	0.00	7.70	5.00	15.79
1.00	5.00	0.00	300.00	7.70	25.00	1.25
1.00	5.00	150.00	0.00	5.70	5.00	0
1.00	5.00	150.00	0.00	7.70	25.00	3.83
1.00	5.00	150.00	300.00	5.70	25.00	4.75
100.00	1.00	0.00	0.00	7.70	5.00	10.52
100.00	1.00	0.00	300.00	5.70	5.00	13.06
100.00	1.00	0.00	300.00	7.70	25.00	0.83
100.00	1.00	150.00	0.00	7.70	25.00	2.55
100.00	1.00	150.00	300.00	5.70	25.00	3.17
100.00	5.00	0.00	300.00	7.70	5.00	15.79
100.00	5.00	150.00	0.00	7.70	5.00	0
100.00	5.00	150.00	300.00	5.70	5.00	0
1.00	1.00	0.00	0.00	5.70	25.00	1.53
1.00	1.00	0.00	0.00	7.70	5.00	10.52
1.00	1.00	0.00	300.00	5.70	5.00	13.06
1.00	3.00	75.00	150.00	6.70	15.00	3.98
100.00	3.00	75.00	150.00	6.70	15.00	3.98
10.00	1.00	75.00	150.00	6.70	15.00	3.34
10.00	5.00	75.00	150.00	6.70	15.00	5.02

10.00	3.00	0.00	150.00	6.70	15.00	2.46
10.00	3.00	150.00	150.00	6.70	15.00	7.52
75.68	1.55	55.60	5.10	6.68	6.70	10.67
12.37	3.77	115.90	90.40	6.20	12.50	8.4
2.63	1.14	4.30	292.40	7.06	20.50	7.41
6.22	1.92	119.00	0.90	5.71	10.60	8.28
16.41	2.57	31.80	95.80	6.60	13.90	3.25
19.15	2.43	43.00	161.80	7.09	7.80	8.41
2.86	4.96	36.40	66.60	6.53	5.40	16.05
49.20	3.51	104.00	101.90	7.23	17.10	4.64
3.91	3.62	140.40	48.20	7.37	21.50	2.81
3.09	3.99	102.90	70.10	7.57	8.90	12.45
1.65	1.80	28.50	63.40	7.08	16.50	2.19
4.94	4.92	139.30	150.50	6.63	9.50	17.75
17.95	3.83	44.30	198.00	6.09	20.90	2.11
17.11	1.29	75.00	293.00	6.01	8.40	9.36
25.46	4.35	40.40	138.30	6.48	8.20	10.12
28.26	4.52	72.50	114.20	5.91	23.30	2.33
2.49	3.35	19.80	29.50	5.77	11.00	5.51
37.68	2.33	89.20	247.60	6.46	20.00	2.57
3.64	4.20	147.60	166.60	7.60	7.30	25.49
9.40	1.58	13.50	140.50	6.90	15.30	2.24
6.83	2.98	39.40	84.90	7.30	5.20	15.12
76.59	4.19	81.90	171.70	6.84	15.30	4.58
1.21	1.76	64.30	266.20	6.04	13.40	4.28
11.82	2.72	100.40	263.60	6.57	8.60	11.52
1.52	3.85	5.70	297.30	7.25	13.20	3.39
71.90	2.22	54.00	132.30	5.88	15.10	3.54
60.02	2.19	2.30	223.30	7.60	6.00	9.25
33.25	3.74	11.50	173.50	7.21	17.40	2.17

36.63	2.43	65.90	68.90	7.42	14.90	3.51
7.26	3.47	79.80	214.00	5.75	8.00	13.9
2.56	2.55	34.00	50.80	5.90	19.30	2.08
19.97	2.50	110.00	254.00	7.46	22.70	2.39
6.58	4.23	22.00	206.40	7.51	13.80	3.62
14.50	2.38	67.30	227.30	6.82	10.80	6.05
2.25	3.29	112.60	34.50	6.97	10.30	10.05
15.31	4.69	25.50	297.50	7.10	10.00	6.61
1.92	4.53	84.60	110.30	6.42	23.70	2.02
2.40	2.06	29.40	272.00	7.03	24.90	1.45
1.10	1.96	128.50	45.80	7.13	17.20	4.36
1.21	2.96	69.90	37.60	7.66	6.30	14.32
32.33	1.49	149.20	217.40	6.11	18.60	4.79
52.67	1.11	136.30	232.60	7.15	11.80	8.19
10.03	4.60	9.10	169.60	6.72	24.30	1.34
30.44	3.66	108.50	204.00	7.00	16.60	4.61
51.20	1.35	18.60	16.00	6.00	15.80	2.34
5.11	1.80	59.00	157.10	6.69	18.40	2.26
1.97	1.19	109.50	105.90	5.82	18.30	3.62
56.07	3.68	143.20	136.80	6.19	10.90	13.32
23.63	2.69	8.00	236.90	6.65	6.90	8.41
45.18	1.71	121.30	251.50	6.41	22.10	2.67
21.68	2.64	91.00	103.50	7.56	5.60	19.06
4.29	2.53	121.20	122.20	7.44	19.00	3.61
98.42	3.04	62.70	56.90	6.56	5.70	16.1
20.90	2.86	96.30	144.50	6.02	19.70	3.12
1.78	3.72	53.50	13.80	7.64	15.90	3.29
40.12	4.59	20.60	279.40	6.53	21.00	1.87
1.15	3.49	83.30	259.60	5.94	8.80	11.56
13.17	2.92	97.50	164.90	7.38	17.90	3.41

96.09	4.78	134.70	3.30	7.02	10.10	14.96
46.92	4.65	146.40	85.00	5.92	11.90	14.18
2.73	4.83	0.10	10.80	6.77	14.20	3.31
69.39	1.08	141.60	262.30	7.24	16.80	4.77
5.43	1.89	58.50	230.10	5.84	22.00	1.88
43.61	1.24	106.50	276.10	6.60	23.00	2.08
2.07	3.38	45.60	274.80	6.29	6.60	12.47
64.79	4.73	125.40	22.80	5.74	7.60	24.61
1.30	4.44	61.00	109.60	5.86	14.60	4.99
57.56	3.18	88.30	246.90	7.27	21.50	2.37
8.15	2.07	10.10	187.80	6.07	8.00	6.92
16.88	1.99	51.20	154.30	6.44	18.90	2.1
10.84	4.27	77.30	54.50	6.87	12.60	6.17
15.12	3.58	112.20	209.00	7.34	24.60	2.35
19.84	2.90	105.50	194.40	7.63	24.40	2.1
3.22	2.78	70.50	117.10	7.54	18.70	2.54
86.02	4.43	38.00	19.50	5.97	14.80	4.04
2.10	3.89	32.90	89.60	6.05	16.00	3.16
8.69	2.01	124.20	228.20	6.92	12.90	6.89
12.87	2.75	131.90	8.80	6.27	18.10	4.63
84.57	1.44	75.00	133.40	6.18	19.60	2.29
4.79	1.06	94.50	184.40	6.95	24.00	1.7
6.05	2.16	92.60	159.40	6.32	17.00	3.49
62.47	4.32	87.10	31.70	6.38	12.10	7.33
4.42	1.66	17.40	211.50	7.49	20.20	1.42
4.02	3.10	131.10	191.20	6.40	12.70	8.53
1.05	3.90	35.70	35.20	6.72	22.70	1.63
27.63	4.17	126.40	98.30	6.65	13.00	8.69
2.98	3.22	41.50	269.00	6.50	23.20	0.0188
1.45	1.42	67.60	284.40	6.79	10.40	5.93

4.63	1.69	135.10	289.50	6.31	9.50	12.47
1.59	3.30	7.50	123.50	5.82	21.80	1.59
35.27	4.10	114.80	21.40	6.75	11.40	9.59
26.21	1.62	14.80	80.90	7.15	23.40	1.08
5.73	2.82	117.60	62.30	7.29	6.40	20.13
8.33	3.11	48.40	177.60	6.81	23.70	1.5
3.53	2.63	77.70	244.20	6.74	16.20	3.43
5.33	1.37	138.60	42.10	6.26	14.40	6.45
41.64	4.47	91.90	175.20	7.05	14.10	5.84
24.60	3.55	71.30	186.70	5.79	21.20	2.62
1.54	3.01	95.00	239.10	7.17	11.30	7.28
1.32	4.83	51.60	126.70	7.53	7.30	12.7
89.99	3.24	16.00	145.40	6.14	17.80	2.18
9.18	4.40	24.40	285.90	7.40	24.10	1.43
7.78	2.30	26.90	219.30	5.72	19.50	2.07
22.41	3.95	117.20	256.20	6.15	9.10	14.47
6.37	4.12	56.40	195.40	7.18	15.60	3.64
1.38	1.83	61.50	180.40	6.90	9.80	6.43
10.70	1.03	99.90	242.00	5.96	20.50	2.34
3.71	4.99	143.90	94.70	7.70	9.30	19.14
3.39	2.12	30.10	281.20	7.67	22.50	1.32
9.69	1.31	3.50	25.80	6.93	20.10	1.29
66.48	3.42	23.60	58.30	6.86	17.60	2.23
38.76	2.36	80.20	79.60	6.22	12.30	5.62
13.67	4.70	123.00	221.20	7.37	22.30	3.51
11.36	4.03	86.20	147.70	6.34	11.60	7.56
1.73	4.05	49.60	76.00	7.33	13.60	4.33
7.36	4.89	132.70	72.90	6.36	5.00	0
29.88	2.24	102.00	44.70	6.50	6.00	18.77
82.28	3.16	47.20	200.50	6.24	7.10	11.17

1.02	1.52	145.90	119.30	7.48	24.70	2.61
2.21	1.20	129.50	129.50	6.13	20.70	3.2

Table S4. Additional combination of PMP.

**Temperature (°C)**	**pH**	**NaCl (%)**	**Nitrite (ppm)**	**Initial concentration (log CFU/g)**	**t_{100} (h)**
10.00	5.00	1.00	0.00	1.00	198.97
30.00	7.00	5.00	140.00	5.00	11.7
10.00	5.00	1.00	140.00	5.00	487.39
10.00	5.00	5.00	0.00	5.00	472.34
10.00	5.00	5.00	140.00	1.00	1093.49
10.00	7.00	1.00	0.00	5.00	50.56
10.00	7.00	1.00	140.00	1.00	56.67
10.00	7.00	5.00	0.00	1.00	79.48
30.00	5.00	1.00	0.00	5.00	17.9
30.00	5.00	1.00	140.00	1.00	44.51
30.00	5.00	5.00	0.00	1.00	47.1
30.00	7.00	1.00	0.00	1.00	5.74
10.00	5.00	1.00	0.00	5.00	199.1
10.00	5.00	1.00	140.00	1.00	481.85
10.00	5.00	5.00	0.00	1.00	454.6
10.00	5.00	5.00	140.00	5.00	1136.67
10.00	7.00	1.00	0.00	1.00	51.02
10.00	7.00	1.00	140.00	5.00	57.68
10.00	7.00	5.00	0.00	5.00	79.4
10.00	7.00	5.00	140.00	1.00	89.4
10.00	5.00	3.00	70.00	3.00	83.8
30.00	7.00	3.00	70.00	3.00	36.58
10.00	7.00	3.00	70.00	3.00	64.19
30.00	5.00	3.00	70.00	3.00	43.3
20.00	6.00	1.00	0.00	3.00	16.32
20.00	6.00	1.00	140.00	3.00	27.1

20.00	6.00	5.00	0.00	3.00	32.2
20.00	6.00	5.00	140.00	3.00	53.4
10.00	6.00	1.00	70.00	3.00	89
10.00	6.00	5.00	70.00	3.00	153.9
25.60	5.06	1.49	14.10	3.20	26.9
14.10	6.78	2.64	79.50	1.26	35.26
28.40	5.00	4.70	114.70	3.60	102.7
19.20	5.57	2.18	49.30	4.67	57.77
10.50	6.91	2.12	135.90	3.55	58.3
25.50	5.46	4.51	18.60	3.65	30.8
22.30	6.85	1.32	4.20	4.83	10.69
19.50	5.49	1.08	118.80	2.68	48.67
26.90	5.51	4.63	89.10	2.63	39.9
26.40	6.99	1.10	117.70	1.12	8.27
15.40	6.57	3.91	45.90	1.56	35.8
17.00	6.53	2.05	126.10	2.11	28.31
30.00	5.82	3.60	74.10	3.82	16
13.60	5.78	4.22	22.60	4.36	79.8
20.00	5.20	4.28	22.10	2.56	20
28.90	5.12	2.40	4.80	2.99	23
29.10	5.35	2.63	96.00	2.89	23
12.90	5.59	2.31	62.50	2.02	93.9
18.50	6.76	4.12	113.70	3.50	24.9
22.80	6.62	3.76	27.90	1.81	14.24
28.10	6.24	3.97	13.10	4.55	16.4
29.50	5.81	2.20	43.20	2.51	11.09
16.90	6.50	1.81	83.30	2.24	25.13
27.20	6.26	1.16	111.10	4.42	9.8
25.10	6.01	3.56	75.40	3.59	19.1
29.70	6.89	1.87	101.50	4.63	6.98
22.50	6.81	1.84	87.40	3.04	12.09
23.70	6.05	1.20	42.70	4.25	12.27
21.10	6.55	1.78	11.30	4.96	12.89

13.80	5.61	3.23	121.80	1.29	127.89
10.20	6.83	3.44	38.90	3.99	64.63
26.70	5.79	1.60	132.60	1.71	18.38
24.00	6.30	1.46	33.60	4.60	10.78
11.10	6.57	4.95	58.50	2.37	81.1
24.60	6.14	2.02	97.80	3.88	15.17
26.00	5.76	1.26	68.80	1.21	14.19
23.40	6.71	3.19	54.90	1.74	12.99
17.40	6.43	2.46	84.60	4.76	27.2
22.00	5.56	3.29	79.30	3.35	47.6
22.60	6.33	3.13	124.40	3.69	20
12.00	6.93	4.36	11.80	3.44	54.61
10.70	5.10	2.30	34.60	1.05	233.25
20.80	6.83	2.77	19.20	3.25	14.93
18.30	6.23	3.68	28.50	3.75	28.65
11.80	6.28	3.08	82.50	4.03	67.08
24.80	5.16	2.72	139.90	1.89	67.2
18.90	5.33	4.26	112.70	3.01	128.9
15.30	5.34	2.94	40.50	3.95	100.5
24.50	5.83	3.06	109.50	2.09	26.1
25.30	5.19	4.03	90.80	4.97	24
20.90	6.08	1.02	31.00	1.31	15.3
24.20	5.66	3.38	2.50	2.57	124
17.80	5.74	3.85	134.40	4.04	20
20.20	5.17	3.40	37.00	1.37	69
22.90	6.78	2.88	71.90	2.42	13.23
23.30	5.38	1.95	24.70	1.80	26.06
14.30	6.40	3.32	130.30	2.30	51.09
23.10	5.72	1.69	110.10	4.72	26.5
14.90	6.18	2.08	119.00	4.16	45.3
11.20	5.40	2.38	93.50	3.93	187
28.00	6.96	2.85	107.20	4.51	8.85
19.90	6.93	4.56	41.70	1.17	21.84

19.10	6.72	1.23	69.20	1.94	16.46
18.30	5.26	2.93	76.70	1.42	82.8
27.70	5.22	1.04	51.90	1.08	22.2
10.10	5.41	1.91	98.60	3.21	214.2
20.60	6.61	4.92	48.10	1.92	23.31
19.80	5.15	4.76	35.40	2.15	97.5
16.60	6.15	3.98	57.30	1.84	40.4
29.40	6.02	4.19	116.10	3.43	18
12.10	6.59	3.25	81.10	4.18	54.2
21.50	5.48	4.03	133.60	2.72	72
11.50	5.88	3.72	20.10	1.36	88.3
17.90	6.09	2.50	91.40	1.01	32.54
21.90	5.65	2.49	66.00	2.20	34.8
28.70	5.04	2.75	60.50	1.15	42.7
13.80	5.45	4.58	129.40	3.52	
18.70	6.68	1.40	102.80	2.05	18.6
13.40	6.98	3.80	72.80	2.47	43.35
26.30	5.52	4.79	92.90	4.13	45
16.40	5.62	1.50	23.90	2.94	42.4
12.80	5.67	2.54	15.90	1.58	71
28.70	6.32	2.99	138.40	2.84	12.31
25.80	6.34	3.66	64.70	4.77	13.78
17.30	6.19	1.43	53.10	2.80	24.84
21.80	6.38	3.01	54.60	4.09	16.6
16.10	5.90	4.15	67.00	2.74	61
12.50	6.88	4.86	77.40	4.30	58.87
20.40	6.06	3.49	123.20	1.44	32.6
27.20	5.91	3.36	44.60	3.08	15.4
12.20	6.46	3.78	105.00	4.86	64.7
13.10	5.99	2.82	100.60	4.49	73.1
14.40	5.95	2.69	127.50	1.68	79.6
21.50	6.21	1.70	137.10	3.83	20.6
11.00	5.92	4.98	38.10	2.65	86.1

23.50	6.69	1.28	85.20	2.28	10.8
15.90	5.70	4.90	6.80	3.33	70.9
13.20	6.74	3.58	32.50	4.37	43.5
26.70	6.10	4.63	0.60	3.72	14.6
11.50	6.48	1.77	26.10	3.29	72.6
24.10	5.30	2.16	8.60	4.30	26.1
24.80	6.36	4.81	88.30	3.38	18.8
27.50	5.94	2.59	63.30	2.36	13.66
14.70	5.27	4.30	56.60	2.83	160.8
26.10	6.51	2.26	131.60	1.66	11.58
12.30	5.03	1.65	1.40	1.61	155.7
28.20	6.64	3.16	125.20	3.16	10.34
15.50	5.42	3.50	99.70	4.91	130.1
16.70	6.39	4.40	71.20	3.11	38.7
15.10	5.87	4.47	120.90	1.98	92.2
18.00	6.12	2.35	7.40	1.49	24.23
21.20	5.09	4.40	108.10	2.44	143.3
29.20	5.98	1.35	30.10	4.89	8.08
16.20	5.71	4.68	61.50	2.19	82.8
27.40	5.08	1.62	16.50	2.91	23.77
10.60	5.54	3.90	94.80	4.44	238.4
14.60	5.25	1.56	105.10	1.51	124.7
17.70	6.67	4.50	47.10	4.65	28
15.70	5.28	4.09	10.40	4.22	170
19.70	6.42	1.98	51.20	3.80	17.9
